# Physical oceanographic factors controlling the ocean circulation-induced magnetic field

**DOI:** 10.1098/rsta.2024.0076

**Published:** 2024-12-02

**Authors:** David S. Trossman, Robert H. Tyler, Helen R. Pillar

**Affiliations:** ^1^ Cooperative Institute for Satellite Earth System Studies (CISESS)/Earth System Science Interdisciplinary Center (ESSIC), University of Maryland, College Park, MD, USA; ^2^ NASA Goddard Space Flight Center, Planetary Magnetospheres Laboratory, Greenbelt, MD, USA; ^3^ University of Texas at Austin, Oden Institute for Computational Engineering and Sciences, Austin, TX, USA

**Keywords:** ocean, circulation, magnetic, ECCO, EM-APEX, conductivity

## Abstract

Oceanic tidal constituents and depth-integrated electrical conductivity (ocean conductivity content, or OCC) extracted from electromagnetic (EM) field data are known to have a strong potential for monitoring ocean heat content, which reflects the Earth’s energy imbalance. In comparison to ocean tide models, realistic ocean general circulation models have a greater need to be baroclinic; therefore, both OCC and depth-integrated conductivity-weighted velocity (${𝐓}_{𝛔}$) data are required to calculate the ocean circulation-induced magnetic field (OCIMF). Owing to a lack of ${𝐓}_{𝛔}$ observations, we calculate the OCIMF using an ocean state estimate. There are significant trends in the OCIMF primarily owing to responses in the velocities to external forcings and the warming influence on OCC between 1993 and 2017, particularly in the Southern Ocean. Despite being depth-integrated quantities, OCC and ${𝐓}_{𝛔}$ (which primarily determine the OCIMF in an idealized EM model) can provide a strong constraint on the baroclinic velocities and ocean mixing parameters when assimilated into an ocean state estimation framework. A hypothetical fleet of full-depth EM-capable floats would therefore help improve the accuracy of the OCIMF computed with an ocean state estimate, which could potentially provide valuable guidance on how to extract the OCIMF from satellite magnetometry observations.

This article is part of the theme issue ‘Magnetometric remote sensing of Earth and planetary oceans’.

## 1. Introduction

One objective of this study is to use an ocean state estimate to provide a description of the long-term (25 years) behaviour of the measurable variables that primarily determine temporal variability in the ocean circulation-induced magnetic field (OCIMF). For this objective, separating the steady component of the OCIMF from crustal/lithospheric anomalies is unnecessary. A second objective of this study is to use an ocean state estimation framework to explore the constraints that potential measurements of variables which primarily determine the variability of the OCIMF may offer on the physical ocean state.

Because the ocean is electrically conductive, magnetometry data can provide information about physical oceanographic variables, including ocean heat content (OHC) [1], waves such as tsunamis [2,3] and state variables like the ocean circulation fields [4]. The electrical conductivity of the ocean arises from the ionic composition of seawater [5]. As ocean currents and tides transport these ions through the Earth’s magnetic field, secondary electric fields and electrical currents (with associated magnetic fields) are generated [6–9]. Previous studies have used theory and some available data to calculate the OCIMF [7,10–12], but little is known about its long-term change over time or the measurable variables that primarily determine it, which we characterize in the present study. The ocean tide-induced magnetic field (OTIMF) has a similar amplitude to the OCIMF, but constituents of the OTIMF are easier to extract from electromagnetic (EM) data owing to the inherent predictability of tidal motions [13–15]. OTIMFs contain information about ocean conductivity content (OCC, also known as conductance) variability and tidal transport [16]. Thus, constituents of the OTIMF can potentially be used to help monitor OCC, which is a good proxy for OHC [1,17–22] owing to the fact that the processes that dominate the heat budget (namely, advection and air–sea flux) also dominate the conductivity budget in most locations [23].

Unlike ocean tidal transports, we cannot calculate ocean general circulation transports from magnetic data owing to the baroclinicity of the general circulation. The baroclinicity of the circulation results in non-vanishing depth integrals when ocean velocities are weighted by conductivities, which are required to calculate the OCIMF. We define the OCC (Σ) and depth-integrated conductivity-weighted velocities (${𝐓}_{𝛔}$), as


(1.1) $$\begin{array}{rl} \Sigma & =\int \sigma dz,\\ T_{\sigma } & =(\int \sigma udz)/(\int \sigma dz), \end{array}$$

where the integrals ∫⋅dz are overall depths from the seafloor to the sea surface, σ is the electrical conductivity and 𝐮 is the ocean velocity field. We refer to the numerator of ${𝐓}_{𝛔}$ (∫σ𝐮dz) as ocean conductivity transport (OTC), hereafter. While the accuracy of Σ can be improved by including a contribution owing to sediment conductance, we deliberately do not do this for the present study because we use an idealized case where all electric current is confined to the ocean, as in [7]. Reasons for doing this include (i) the sediment conductance is not known with the same accuracy as the ocean conductance and so including it creates a confounding factor; (ii) the idealized case retains expected oceanographic conservation principles, thereby facilitating the budget analyses; and (iii) the idealized case describes a useful end-member case, as including conductive sediments and the mantle would tend to decrease the strength of the remotely observable (poloidal) magnetic fields.

Magnetometry is similar to gravimetry in at least one sense: it is used to detect signals that are depth-integrals rather than properties at boundaries. The ocean is considered electrically thin at periods much greater than 10 min [24], meaning that the diffusion length/attenuation scale inside the ocean is much smaller than the ocean thickness (i.e. the seafloor depth of the ocean). As a consequence of this, excited horizontal electric fields are approximately depth-independent [7,8]. The *in situ* toroidal component of the OCIMF is highly depth-dependent but does not reach outside the ocean. The poloidal component of the OCIMF, on the other hand, depends on the depth-integrated electric currents and is potentially detectable outside the ocean. This is why the OCIMF depends on Σ and ${𝐓}_{𝛔}$ rather than just sea-surface electrical conductivity and velocities. Unfortunately, this means that monitoring OCIMF is challenging because Σ and ${𝐓}_{𝛔}$ cannot be monitored using remote sensing data that are collected for more conventional physical oceanographic purposes. Only the surface (top centimetre or less) electrical conductivity can be monitored using satellite-derived sea-surface temperature and salinity data [23]. Since the 1960s, there have been eXpendable BathyThermographs deployed that extend down nearly 1000 m along repeated transects, but where only temperature is recorded, not conductivity. Conductivity, temperature and depth (CTD) instruments have been used to monitor conductivity down to the seafloor along repeated transects for decades, but their global coverage has been lacking in spatiotemporal resolution. The improved coverage by the global array of Argo profiling floats enables depth-integrated conductivity estimates in the upper 2000 m throughout ice-free regions. Deep Argo floats that can extend down to 6000 m in ice-free regions are not yet global in coverage. As a result, full-depth estimates of OCC rely on climatological conductivities below 2000 m [25]. In addition to this observational sampling bias problem, a thermodynamic inconsistency results from not using co-observed temperature and salinity to calculate Σ. There are even more pronounced observational sampling issues with monitoring ${𝐓}_{𝛔}$, as there are several surface current [26,27], near-surface current [28] and Argo float-based [29] data products in the upper water column based on observations, but no full-depth velocity datasets are available from observations alone, or even combined with theory. Therefore, we rely on an ocean state estimation framework that produces a well-validated dynamically and kinematically consistent model–data synthesis for estimating global Σ and ${𝐓}_{𝛔}$ (and therefore OCIMF with some assumptions described below) in the present study.

Owing to difficulties with extracting the OCIMF from the observable total magnetic field, previous studies have examined the effect of assimilating model-based OCIMF data on ocean temperature, salinity and velocities [4]. We take a similar approach but assimilate physical ocean variables from an observationally constrained and dynamically consistent ocean state estimation product [30,31] to estimate the OCIMF. Importantly, by using a non-sequential adjoint-based approach for data assimilation, we can assess the constraints which the variables that determine the OCIMF exert on the full ocean state and uncertain model parameters on multi-decadal timescales. Specifically, the adjoint can be used to obtain sensitivity maps showing how any quantity of interest, including OCIMF data, changes with the model variables. Examining how these maps evolve through space and time reveals the dynamical signals sequestered in the OCIMF acquisitions [32]. Furthermore, the dynamic and kinematic consistency of the state estimate allows us to perform meaningful budget analyses [33,34] to elucidate the underlying drivers of multidecadal trends in the variables that primarily determine the OCIMF in an idealized EM modelling framework (Σ and ${𝐓}_{𝛔}$).

We supplement the output of the ocean state estimate with an idealized EM model similar to that of [7] in which all electric current circulates within the ocean and electric charge is conserved exactly to examine the long-term behaviour of the OCIMF. In this initial analysis, we use this idealized model to provide a baseline case that avoids conflation with the relatively larger uncertainties in the database for sediment and mantle conductivity. In addition, keeping the sediment conductivity out of the conductivity weighting of the velocity makes the oceanographic balance analyses clearer (e.g. the convergence of conductivity transport is no longer balanced by its ocean sources/sinks if the conductivity transport includes weighting by sediment conductance). This model approach simplifies these initial analyses and also provides a baseline that can be regarded as an upper estimate of the OCIMF amplitudes because we expect that including sediment and mantle conductivity will have the tendency to decrease the remotely observable (poloidal) magnetic field while increasing the toroidal magnetic field at and below the seafloor. Physically, the electric potentials generated via the flow-generated Lorentz forces are partly relieved by the shorting of electric currents through the seafloor paths.

This article is organized as follows: we first describe the model for calculating the OCIMF at the sea surface and the adjoint-based ocean state estimate in §2. In §3, we present time series of the inputs and outputs to the OCIMF model over a period spanned by the ocean state estimate (1993–2017)—longer than any period assessed in the existing literature—and reveal significant trends in OCC, OCT and the OCIMF. We then explore the forcings underpinning these trends via an analysis of ${𝐓}_{𝛔}$ and a momentum budget as well as the potential constraint EM data could exert upon assimilation into the ocean state estimation framework, via adjoint-based sensitivity analysis. We conclude with a summary, caveats and suggested future directions.

## 2. Methods

We use the Estimating the Circulation & Climate of the Ocean (v.4 release 4, ECCOv4r4, hereafter) ocean state estimate [30,31] to generate the data for our analyses. We use ECCOv4r4 for the following reasons: (i) it uses the MITgcm configured at 1° horizontal resolution and runs in a dynamically and kinematically consistent manner; (ii) it has been extensively constrained by *in situ* and satellite data; (iii) it spans a 26-year period (1992–2017); (iv) it has been extensively used for studies of ocean variability; and (v) it has an adjoint capability that has been exploited to understand origins of variability and observation effects. This ocean state estimate and the EM model are described in more detail in the appendix. We perform budget analyses and sensitivity calculations using ECCO to better understand the trends that we find with the EM model, all of which are described below.

### (a) EM model

Forward model calculations of the magnetic fields generated by large-scale ocean circulation [7–9,12,35–38] require prescription of the ocean flow, the Earth’s main magnetic field, ocean electrical conductivity and data or assumptions regarding electrical conducting material outside the ocean (the mantle, sediments and continents). In studies so far, ocean flow has come from ocean models (as described above), and the Earth’s main magnetic field is readily obtained from geomagnetic field models. The ocean conductivity used in modelling has evolved from idealized assumptions (e.g. a constant value [7]) to a heterogeneous field calculated from the temperature and salinity data of the same ocean model providing the flow [8]. Note that climatological ocean conductivity data have become available more recently [25,39] but have only been used in estimating magnetic fields of the barotropic tides.

The large-scale ocean can be regarded as a relatively good electrical conductor sandwiched between relative insulators. In early studies [7,8] no conductors outside the ocean were included. It is expected that model accuracy increases with the addition of conducting wet sediments [36], a conductive mantle [9] and even the highly conducting Earth’s core [38]. It should be noted, however, that the prescription of conductivity in the regions outside the ocean has a much higher level of uncertainty than the uncertainty in assigning the ocean conductivity. While ocean conductivity varies over a small range in the ocean and is largely predictable, conductivity estimates can vary by orders of magnitude in the sediments, mantle and land [40–42]. The thicknesses of these layers are also much less certain than the ocean thickness. Importantly, there is general agreement in large-scale magnetic field estimates from simple models excluding conductive regions exterior to the ocean [7] and more complex models that include them. The differences in results so far appear to owe more to the different ocean circulation models used and their resolution rather than differences in exterior conductivity assumptions. Theoretical [24] and numerical [12] studies have described differences when a radially varying mantle conductivity is added, but the realistic mantle is highly three dimensional and includes local features such as wet subducting slabs and spreading ridges. The added realism in including these external conductors is currently hard to quantify and comes at a cost of convolving the large uncertainties of the exterior conductors with the much better-known ocean conductivity.

As described above, for the purposes of the modelling in this study, we return to the idealized assumptions of [7] where the external regions are treated as insulators. In this simple model, all electric current remains within the ocean. This is advantageous in simplifying the conductivity budget, allowing causal forcings underpinning content/transport trends to be more easily investigated. As this study is primarily concerned with the temporal variability in the OCIMF, when external conductors are included, one might require the need to consider the temporal variability of the conductivity in these regions.

### (b) Conductivity and momentum budget analyses

Our idealized EM approach aims to best conserve the dynamical properties of the ocean, which we intend to understand by using budget frameworks. Drivers of OCIMF trends over the period 1993–2017 are interrogated by inspecting drivers of both conductivity and velocity variations. The ECCO-derived conductivity [23] and momentum [33] budgets are given by


(2.1) $$\begin{array}{ccc} & \begin{array}{c} \rho \frac{d\sigma }{dt}=-\nabla \cdot {𝐉}^{\sigma }+\rho Q^{\sigma }, \end{array} & \end{array}$$

(2.2) $$\partial u_{tot}/\partial t=-(2\Omega \times u_{tot})-(\zeta \times u_{tot})-\nabla KE-(g\rho^{\prime }/\rho_{0})\hat{k}+\nabla \cdot \tau /\rho_{0}-\nabla p/\rho_{0},$$

respectively, where $d/dt=\partial /\partial t+({𝐮}_{𝐭𝐨𝐭})\cdot \nabla$ is the material derivative, ${𝐮}_{𝐭𝐨𝐭}=𝐮+{𝐮}^{*}$ is the total velocity field, 𝐮 is the resolved velocity field, ${𝐮}^{*}$ is the parameterized eddy-induced velocity that represents unresolved motions, ρ is the locally referenced potential density, ${𝐉}^{\sigma }$ are the fluxes associated with the parameterized mixing along constant density surfaces (‘along-isopycnal diffusivities’) and across constant density surfaces (‘diapycnal diffusivities’) for electrical conductivity, $Q^{\sigma }$ are the sums of sources and sinks of electrical conductivity, 𝛀 is Earth’s rotation rate vector, 𝛇 is the vorticity vector, KE is the kinetic energy, $g=9.806\;m^{2}s^{-1}$ is the acceleration due to gravity, $\rho_{0}$ is a reference density, 𝛕 is the external stress (such as that owing to winds or dissipation), p is the pressure and $\hat{𝐤}$ is the unit vector away from the centre of the earth. The conductivity budget equation can be broken into potential temperature and salinity contributions [23]. If sediment conductivity were included in our approach, then the budgets in equations (2.1) and (2.2) would no longer be consistent with the EM model.

### (c) Sensitivity analysis and assimilation experiments

We perform a short assimilation experiment to explore the potential constraints offered by observing Σ and $|{𝐓}_{𝛔}|$, which are the primary measurable variables that uniquely determine OCIMF variations using our EM formulation (aside from the small secular variation in the main magnetic field). In our experiment, ECCOv4r4 fields of Σ and $|{𝐓}_{𝛔}|$ from 2005 to 2006 are assimilated at every grid point over the period 1992–1993. Although our specific choice of years is arbitrary, this design ensures conductivity states during the data acquisition and assimilation periods are sufficiently different. Our experiment thus tests the information that large-scale EM field observations could provide from, for example, a global fleet of EM-APEX floats.

## 3. Results

### (a) Distribution and variability of $b_{r}$, OCT and OCC

**Figure 1.**
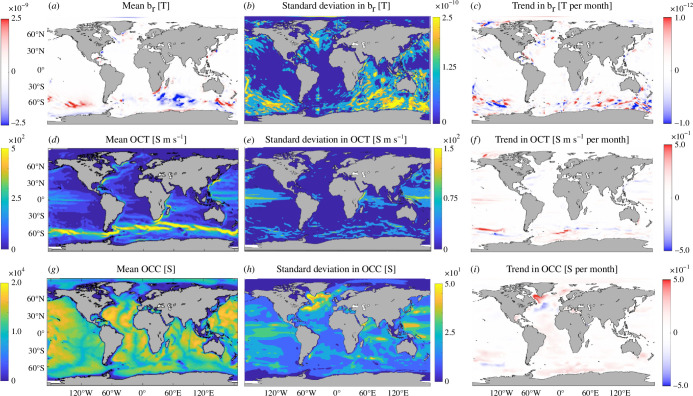
ECCOv4r4-derived (*a*–*c*) OCIMF radial component ($b_{r}$), (*d*–*f*) OCT magnitude and (*g*–*i*) OCC. For each field, we show (*a*,*d*,*g*) the mean, (*b*,*e*,*h*) standard deviation and (*c*,*f*,*i*) trend per month over the period 1993–2017. Units are given in the figure.

**Figure 2.**
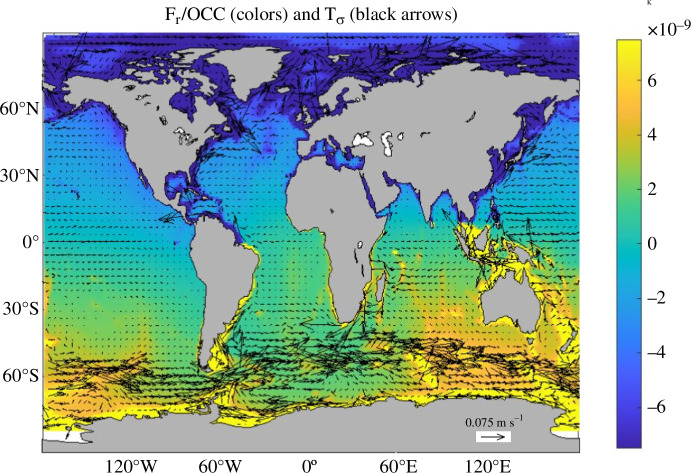
The ratio of the radial component of the background main magnetic field ($F_{r}$) to OCC and ${𝐓}_{𝛔}$ laid on top (black arrows—scale for 0.1 S m s${,}^{-1}$ shown over Antarctica) are shown; ${𝐓}_{𝛔}$ is subsampled every three grid points in latitude and every four grid points in longitude. Here, the ECCOv4r4-derived OCC and ${𝐓}_{𝛔}$ are averaged over 1993–2017.

We first present the means, standard deviation and trends in monthly averaged OCC, OCT and the radial component of the OCIMF ($b_{r}$) (figure 1). Here, the OCIMF is calculated from the monthly averages of OCC and OCT. We examine OCT instead of ${𝐓}_{𝛔}$ first to assess each depth-integral in ${𝐓}_{𝛔}$ independently; $b_{r}$ has a similar amplitude to the horizontal components of the OCIMF (not shown) and is comparable to that used in previous published studies [10,11]. OCT and $b_{r}$ are largest over the Southern Ocean and western boundary regions, where fast currents are found. This is expected because $b_{r}$ is generated where conductivity transport converges and/or flows across isolines of $F_{r}$/Σ, where $F_{r}$ is the radial component of the background main magnetic field. To demonstrate this, we also present $F_{r}/\Sigma$ with ${𝐓}_{𝛔}$ (qualitatively similar to OCT) laid on top (figure 2). Strong ${𝐓}_{𝛔}$ flows from the Antarctic Circumpolar Current (ACC) go across $F_{r}/\Sigma$ contours and generate the two large cells of opposite sign (tilted dipolar magnetic main field) in the Southern Ocean, which explains the spatial pattern in $b_{r}$ there; ${𝐓}_{𝛔}$ is also amplified in the vicinity of the ocean’s equatorial and western intensified jets, but $F_{r}/\Sigma$ is fairly constant in these regions, which is why $b_{r}$ is generally not as large outside the Southern Ocean; Σ is qualitatively comparable to the model’s bathymetry, as has been established in previous studies [22]. This is expected because the spatial variations in Σ are controlled more strongly by changes in water-column thickness than by variations in depth-averaged conductivity. The tendencies in the electrical conductivity budget have been explored in previous work [23], which revealed the dominant roles of surface heat fluxes and advection of temperature and salinity. The tendencies in the other variable affecting OCT (the velocity field), however, have not yet been examined in ECCOv4r4; we return to this later in the present study. Over the 26-year-long simulation, the trends in the three variables shown here are statistically significant with amplitudes comparable to their respective standard deviation (figure 1).

### (b) Relationships between $b_{r}$, OCT and OCC

**Figure 3.**
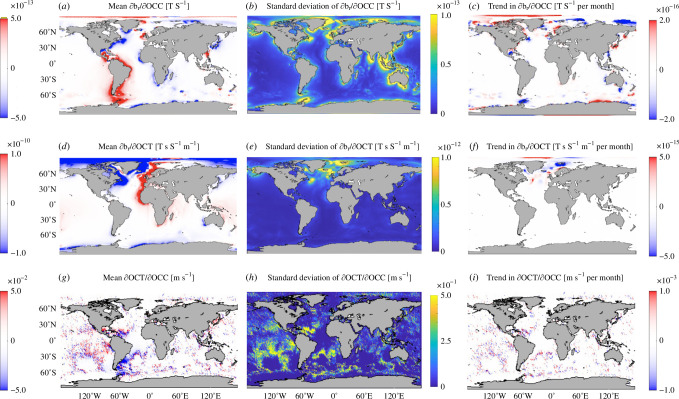
(*a,d,g*) Time-mean and (*b–c,e-f,h–i*) time-varying relationships between the three EM variables $b_{r}$, OCT and OCC, as indicated in each panel heading. The linear trends in these derivatives are statistically significant to the 95% level wherever shown to be non-zero. Positive (red) time-mean sensitivities indicate that (*a*) globally integrated OCIMF increases with increasing OCC, (*d*) globally integrated OCIMF increases with increasing OCT and (*g*) globally integrated OCT increases with increasing OCC. Standard deviation and long-term (1993–2017) trends of these sensitivities are shown in panels (*b,e,h*) and (*c,f,i*), respectively. The noisy appearance is an artefact of corrugation in the contours.

To determine the dependencies between and $b_{r}$, OCT and OCC we computed the partial time-derivatives of each of these three fields, as shown in figure 1, and then calculated the quotients of the same derivatives. The errors in calculating the OCIMF (𝐛) from the monthly averages of OCC and OCT can be inferred from figure 3 after scaling $\partial b_{r}/\partial OCT$ and $\partial b_{r}/\partial OCC$ by the sub-monthly variations in OCC and OCT (approximately a factor of 2 to an order of magnitude smaller than the standard deviation shown in figure 1). The errors associated with using monthly means are at least a factor of 5000 smaller than the magnitude of OCC and at least a factor of 100 smaller than the magnitude of OCT.

The dependencies described by $\partial b_{r}/\partial OCT$ and $\partial b_{r}/\partial OCC$ reveal large-scale spatial patterns and show that where one is large in magnitude, the other is not (figure 3); $\partial b_{r}/\partial OCC$ tends to be largest on the continental shelves because the shelf break introduces large gradients in OCC; $\partial b_{r}/\partial OCC$ tends to be of opposite sign in the northern and southern hemispheres because that is approximately the delineation where 𝐅 changes sign; and $\partial b_{r}/\partial OCT$ on the western and eastern sides of the Atlantic basin tend to be of opposite signs because the velocities tend to be stronger on the western side and with oppositely signed gradients in 𝐮×𝐁. Similar to the $b_{r}$, OCT and OCC fields, over the 26-year-long simulation, the trends in each of the derivative fields lead to changes comparable in magnitude to their monthly averaged standard deviation. Note that the standard deviation of ∂OCT/∂OCC (figure 3*h*) varies more spatially than any other derivative and is often larger than its mean. These results suggest that the timing and locations of data collection for OCT and OCC are important for estimating the (local or global) OCIMF, 𝐛, through constraints on the ocean state estimate. We cannot simply use a climatology of one or both of ${𝐓}_{𝛔}$ or Σ to calculate $b_{r}$, let alone 𝐛.

### (c) ${𝐓}_{𝛔}$ trend attribution

**Figure 4.**
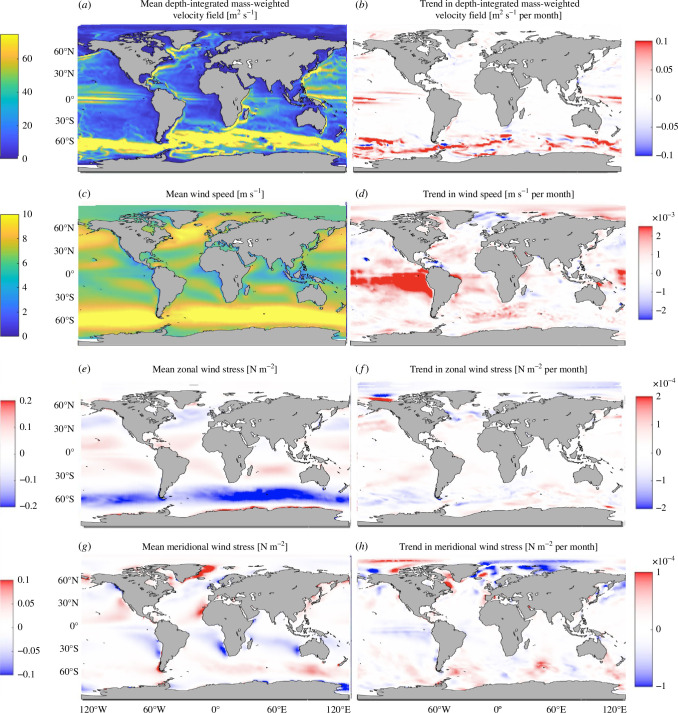
Means (*a,c,e,g*) and trends (*b,d,f,h*) of the depth-integrated mass-weighted velocities (***a,b***—units in (m${,}^{2}$ s${,}^{-1}$)), wind speeds (***c,d***—units in (m s${,}^{-1}$)), zonal wind stresses (***e***,***f***—units in (N m${,}^{-2}$)) and meridional wind stresses (***g***,***h***—units in (N m${,}^{-2}$)). The units for the trends are the same but per month.

**Figure 5.**
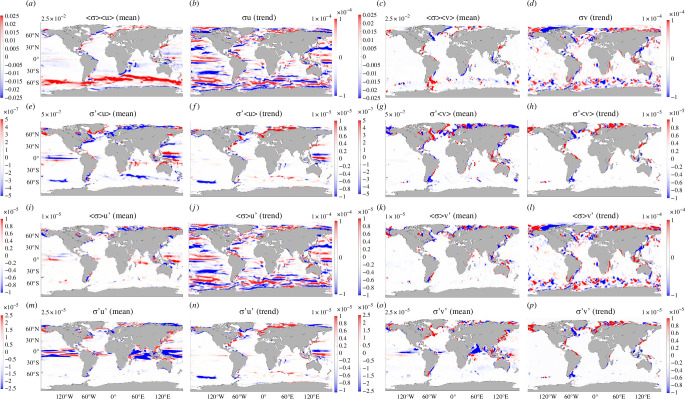
Shown are the means in *a,c,e,g,i,k,m,o* (units in m s${,}^{-1}$) and trends in *b,d,f,h,j,l,n,p* (units in m s${,}^{-1}$ per month) of the terms contributing to ${𝐓}_{𝛔}$ (equation (3.1)) into contributions from average conductivity and average velocity fields (∫<σ><𝐮>dz)/(∫σdz) (***a****,****c***), conductivity anomalies and average velocity fields $\left(\int \sigma^{\prime }<𝐮>dz)/(\int \sigma dz\right)$ (***e***–***h***), average conductivity and velocity anomalies $\left(\int <\sigma >{𝐮}^{\prime }dz)/(\int \sigma dz\right)$ (***i***–***l***) and conductivity anomalies and velocity anomalies $\left(\int \sigma^{\prime }{𝐮}^{\prime }dz)/(\int \sigma dz\right)$ (***m***–***p***). The total trends in ${𝐓}_{𝛔}$ are shown in b,d. Here, the integrals are full-depth, <⋅> is a temporal average, and $(\cdot {)}^{\prime }$ is an anomaly from the temporal average. ***a,b,e,f,i,j,m,n*** are for zonal velocities, and ***c,d,g,h,k,l,o,p*** are for meridional velocities. There is no corresponding trend for the (∫<σ><𝐮>dz)/(∫σdz) term. The total mean of ${𝐓}_{𝛔}$ is not shown but is visually indistinguishable from *a*,*c*. Colour bar maximum (absolute value of minimum) values are listed.

We next discuss the potential causes of the trends seen in figure 1. While the trend in Σ is primarily due to ocean heat uptake and transport [23], it is not immediately clear what causes the trend in OCT (and ultimately ${𝐓}_{𝛔}$, as demonstrated below), which is most prominent in the vicinity of the ACC and has not been investigated in the existing literature. Although there is a significant trend in the mass-weighted velocity field (figure 4), this is qualitatively distinct from the trend in OCT, for example, showing an opposite sign over large regions of the ACC. Additionally, the depth-averaged velocity field is different from OCT [43]. Therefore, the trend in OCT (and ${𝐓}_{𝛔}$) may be caused by changes in local σ(z) and/or the local ${𝐮}_{𝐭𝐨𝐭}(z)$. We mechanistically understand the sources of variability in σ(z) [23], but not in 𝐮. Mechanisms underpinning these changes can include variations in wind forcing, sea-ice concentration and/or water-column baroclinicity. For example, a wind-forcing shift/trend could alter ${𝐓}_{𝛔}$ through perturbing the Ekman transport. The mean wind distributions and their trends (figure 4) show notable qualitative differences with the depth-integrated velocity fields (figure 4), which are also not closely comparable to patterns of trends in sea-ice concentration (not shown). Lacking a clear explanation of ${𝐓}_{𝛔}$ trends from changes in surface conditions, we turn to inspect whether combinations of averages and/or anomalies of σ(z) and 𝐮 dominate the contributions to ${𝐓}_{𝛔}$ (figure 5). This will motivate whether mechanistically understanding the variability in σ(z) and/or 𝐮 is crucial.

**Figure 6.**
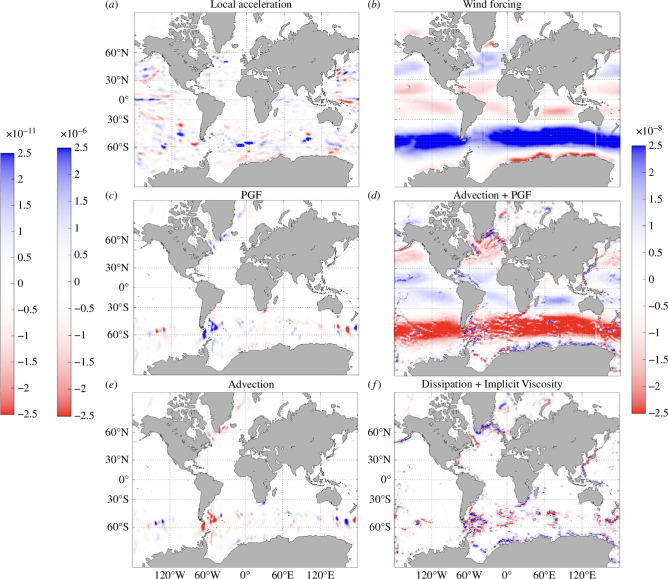
Shown are the depth-integrated terms in the zonal mean momentum budget over 1993–2017 (units in (m${,}^{2}$ s${,}^{-2}$)): (*a*) total plus Adams–Bashforth time-stepping (‘local acceleration’), (*b*) wind forcing, (*c*) surface displacement plus hydrostatic pressure gradient force (PGF), (*d*) PGF plus advection, (*e*) advection and (*f*) dissipation plus implicit viscosity terms. The leftmost colour bar corresponds to *a*. The other colour bar to the left of the maps corresponds to ***c*** and ***e***. The colour bar to the right of the maps corresponds to ***b***,***d***,***f***.

We now break ${𝐓}_{𝛔}$ into its contributions:


(3.1) $$\begin{array}{ccc} & \begin{array}{c} {𝐓}_{𝛔}=\frac{\int <\sigma ><𝐮>dz}{\int \sigma dz}+\frac{\int \sigma^{\prime }<𝐮>dz}{\int \sigma dz}+\frac{\int <\sigma >{𝐮}^{\prime }dz}{\int \sigma dz}+\frac{\int \sigma^{\prime }{𝐮}^{\prime }dz}{\int \sigma dz}, \end{array} & \end{array}$$

where ∫⋅dz is an integral over the full water column, <⋅> is a temporal average and $(\cdot {)}^{\prime }$ is an anomaly from the temporal average. The means and trends of each of these terms for both the zonal (u) and meridional (v) velocity components are shown in figure 5. For each term in equation (3.1), the zonal contribution is larger than the meridional one. The average velocities carrying an average conductivity, <σ><𝐮>/(∫σdz), dominate the average ${𝐓}_{𝛔}$ field distribution. The means and trends of each of the conductivity anomaly terms, $\sigma^{\prime }<𝐮>/(\int \sigma dz)$ and $\sigma^{\prime }{𝐮}^{\prime }/(\int \sigma dz)$, are similar in their spatial distributions except in the eastern equatorial Pacific Ocean. Importantly, the velocity anomalies carrying an average conductivity, $<\sigma >{𝐮}^{\prime }/(\int \sigma dz)$, dominate the trend in ${𝐓}_{𝛔}$ by at least an order of magnitude. Given the importance of the velocity anomalies carrying average conductivity in determining ${𝐓}_{𝛔}$, we next perform a momentum budget in ECCOv4r4 (equation (2.2)) to better understand the mechanisms responsible for variability in 𝐮 (figure 6).

We focus on the zonal component of the momentum budget in ECCOv4r4 (equation (2.2)), as the zonal momentum tendency terms are generally larger than the meridional momentum tendency terms (not shown), and the magnitudes and trends in the zonal contributions to ${𝐓}_{𝛔}$ are also larger than those in the meridional contributions to ${𝐓}_{𝛔}$ (figure 5). The horizontal pressure gradients that cause interior movement of water masses (hydrostatic pressure term, $-\nabla p/\rho_{0}$ in equation (2.2)) and externally forced pressure gradients that cause sea-surface movement (free surface displacement term, $-(g\rho^{\prime }/\rho_{0})\hat{𝐤}$ in equation (2.2)) individually dominate the budget, but partially cancel each other out owing to their opposite signs in each of the zonal and meridional momentum terms, to yield the (surface displacement plus hydrostatic) pressure gradient forces (PGF) tendency term (figure 6). This near-cancellation is probably related to the Sverdrup balance (relating the wind stress curl to depth-integrated meridional transport) on spatial scales larger than several degrees [44] and time scales longer than a few years [45] over the upper ocean. The advection tendency term is very similar to the sum of the PGF tendency terms, which are two orders of magnitude smaller than the individual PGF tendency terms (figure 6). The advection tendency term is composed of a Coriolis component (−(2𝛀×𝐮) in equation (2.2)), vorticity advection (horizontal components of −(𝛇×𝐮) in equation (2.2)), vertical shears (vertical component of −(𝛇×𝐮) in equation (2.2)) and kinetic energy gradients (−∇KE in equation (2.2)). The sum of the advection tendency term and PGF tendency terms is spatially very similar to the wind-forcing tendency term (one of the factors in the $\nabla \cdot 𝛕/\rho_{0}$ term in equation (2.2)). Each of these terms can be important in various sectors of the Southern Ocean, including in the vicinity of the ACC (figure 6). We add the quasi-second order Adams–Bashforth time-stepping [46] tendency term and the total momentum tendency (called ‘local acceleration’ in figure 6*a*) owing to the former being purely numerical and highly correlated (0.84) with (but much smaller than) the total momentum tendency term. The wind-forcing tendency term has a large standard deviation relative to the sum of the advection and PGF terms, except on the eastern side of the subtropical gyres (not shown). We interpret the changes in the depth-integrated velocities to be primarily physically due to the wind, which the advection and PGF terms approximately balance when added together. While the wind-forcing and surface displacement PGF terms only apply in the top layer, the advection and hydrostatic PGF responses to ocean warming [47], and other tendency terms, can occur throughout the water column. Surface forcing and advection are the mechanisms causing the trends in σ(z) [23] and play an important role in the momentum budget, ultimately causing a trend in ${𝐓}_{𝛔}$ because of the dominant role of $\int <\sigma >{𝐮}^{\prime }dz$ in determining OCT (∫σ𝐮dz).

### (d) Constraints provided by $|{𝐓}_{𝛔}|$ and Σ

**Figure 7.**
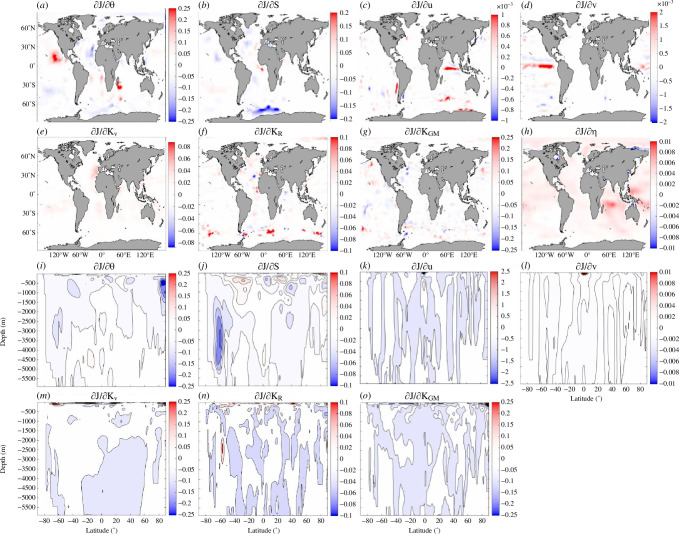
Shown are the (*a–h*) depth-averaged and (*i–o*) zonally averaged ∂J/∂x, where x is (*a,i*) potential temperatures (units in (°C${,}^{-1}$)), (*b,j*) practical salinities (units in (pss${,}^{-1}$)), (*c,k*) zonal velocities (units in (s m${,}^{-1}$)), (*d,l*) meridional velocities (units in (s m${,}^{-1}$)), (*e,m*) vertical diffusivities (units in (s m${,}^{-2}$)), (*f,n*) Redi coefficients (units in (s m${,}^{-2}$)), (*g,o*) Gent–McWilliams coefficients (units in (s m${,}^{-2}$)) and (*h*) sea-surface heights (units in (m${,}^{-1}$)). J is the model–data misfit cost function (equation (A2)) containing contributions only from misfits to synthetic ${𝐓}_{𝛔}$ and Σ data, as described in §2.

Owing to differences in the covariance functions (not shown) in profiles of velocities and σ, those of $|{𝐓}_{𝛔}|$ and Σ and those of the OCIMF with ocean state variables, we anticipate that each of these quantities will provide complementary constraints upon assimilation. Furthermore, the combination of $|{𝐓}_{𝛔}|$ and Σ provides information that ECCOv4r4 does not already receive by assimilating temperature/salinity profiles. To demonstrate this, we plot adjoint-based sensitivities of the combined misfits (equation (A 2)) to synthetic Σ and $|{𝐓}_{𝛔}|$ in our perfectly globally sampled experiment (figure 7). The adjoint sensitivities have an inhomogeneous spatial structure that varies with depth, even though Σ and $|{𝐓}_{𝛔}|$ are depth-integrated quantities. The sensitivities to sea-surface heights (η) are large, indicating that the EM data provides constraint on η variability. This can be understood as follows: η changes because the Σ from future years is used as a constraint, which is larger than the Σ in the model at the time of ingestion (i.e. steric sea-level changes). The cost function is most sensitive to the velocity field near the surface and near the equator, but which component of the velocity field depends on the ocean basin. Velocity response is largest near the surface because of volume conservation after η changes. In ECCOv4r4, there is no specification of how the baroclinic structure should look given a depth-integrated constraint, as there often would be with a sequential data assimilation framework such as that used by [4].

The largest negative adjoint sensitivities to salinity are found where Antarctic Bottom Water mixes with overlying waters to form Circumpolar Deep Water [48,49], and the largest positive adjoint sensitivities to salinity in the Southern Ocean are found where Antarctic Intermediate Water mixes with other water masses in the Pacific sector [50]. Many of the spatial patterns in the adjoint sensitivities to temperature are also found in the adjoint sensitivities to salinity and/or vertical diffusivities, but with opposite sign (e.g. south California Current System and Canary Current), with the exception of the largest (negative) values found in the vicinity of where Atlantic Water can mix with other water masses in the Arctic Ocean when the cold halocline retreats in the Eurasian Basin [51,52]. The largest adjoint sensitivities to the Redi coefficients are at the steering level in the Southern Ocean, where previous studies have found an enhancement of mesoscale eddy stirring [53,54]. Last, the largest adjoint sensitivities to the Gent–McWilliams coefficients are found in regions flanking intensified jets. Aside from the Gent–McWilliams coefficients that are difficult to compare directly with observations, ECCOv4 is known to underestimate the mixing in the above-mentioned regions so an EM-driven adjustment could be in a beneficial direction.

## 4. Discussion

We have investigated the long-term behaviour of Σ, ${𝐓}_{𝛔}$ and the OCIMF from 1993 to 2017 and assessed the physical oceanographic information provided by ${𝐓}_{𝛔}$ and Σ, which together primarily determine 𝐛. There are significant trends in $b_{r}$, OCT and OCC as well as in their dependencies on each other. The trends are due to changes in the external forcing (i.e. wind), which is primarily counteracted by advection and PGFs, and the tendencies in the latter terms are at least in part associated with anthropogenic warming. We performed a preliminary investigation to test the constraints exerted by EM data and suggest it would provide valuable information on ocean mixing. The adjoint sensitivities owing to incorporating information from $|{𝐓}_{𝛔}|$ and Σ are experiment-dependent, but can provide adjustments to the ocean velocities owing to changes in sea-surface heights as well as temperature/salinity in regions where ocean mixing is expected to be important to particular water masses and possibly inaccurately represented (e.g. owing to the use of constant ocean mixing parameters in ECCOv4r4).

From the governing equations, 𝐛 clearly depends on integrals of Σ and ${𝐓}_{𝛔}$. A practical approach for obtaining 𝐛 is to measure $|{𝐓}_{𝛔}|$ and Σ via EM-APEX floats; therefore, an efficient approach is to use $|{𝐓}_{𝛔}|$ and Σ from EM-APEX floats or acoustic doppler current profilers (ADCPs) and CTDs in combination with magnetic data to obtain 𝐛. Calculating 𝐛 requires horizontal gradients in ${𝐓}_{𝛔}$ and Σ so observations alone will never suffice for calculating 𝐛 owing to the density of future EM-APEX floats. In the future, EM data will need to be combined with an ocean general circulation model in a data assimilation framework, to obtain 𝐛. We have now explored this in ECCO and shown time–space complexity in adjoint sensitivities. Given that the adjoint sensitivities with $|{𝐓}_{𝛔}|$ and Σ in ECCOv4r4’s cost function changes as a function of time and space, observing system simulation experiments (OSSEs) should be performed to find out how many and where EM-APEX floats or ADCPs and CTDs are needed for the improved representation of the ECCOv4r4-derived OCIMF. One cursory OSSE-like method to determine the number of EM-APEX-like floats that would be needed to be most cost-effective is to simply subsample and then interpolate Σ and $|{𝐓}_{𝛔}|$ to compute the root-mean-square error relative to the original Σ and $|{𝐓}_{𝛔}|$ fields. Using this method, we find that 45 000 floats are needed to reduce the per cent root-mean-square error to be within 30% for $|{𝐓}_{𝛔}|$ and within 10% for Σ, but considering how there are only 1200 planned Deep Argo floats, it is unrealistic to expect such a large number to be deployed. Dynamically and kinematically consistent spatiotemporal interpolators such as ECCO can help minimize the number of EM-APEX-like floats needed to accurately reconstruct 𝐛. Thus, one strategy for extracting 𝐛 from observations is to assimilate as many EM-APEX-like floats as possible with an ocean state estimate to get a first guess of 𝐛. Leading modes of variability in this first-guess estimate can then be used to help extract 𝐛 from satellite magnetometry data.

In addition to OSSEs, there are many other future directions with overlap between magnetometry and oceanography. One possibility that could continue from our present preliminary study is to explore other valuable information that EM data may contain about the evolving ocean state and the extra value it may add to existing observing networks/conventional observed variables. We have opted for the idealized EM model that can be used for comparison in more sophisticated future studies. A second research direction could be to examine how a higher-resolution ocean model simulation affects the OCIMF. On one hand, the velocities will be larger in eddy-resolving simulations of the ocean [55]. On the other hand, Σ is spatially smooth to the resolution of the World Ocean Atlas climatology [25]. Another option is to use EM field data (from satellite, land, seafloor, ocean or airborne magnetometers) to remotely monitor ocean salt content (OSC) in high-latitude regions because OCC is a proxy for OSC there [21]. The inferred OSC can then be used to derive halosteric sea level and remove its contribution from the steric sea-level estimates in a previously established method to remotely monitor OHC [56]. One final future potential application of using EM data is to constrain biogeochemical models, specifically with total alkalinity because that variable reflects the net charge of the ocean and is strongly correlated with salinity. The correlation between total alkalinity and salinity is 0.77 in the GLODAPv2 database [57], which together with nitrate is an established proxy for total alkalinity [58]. This application could have implications for monitoring the changing marine carbon inventory. Future studies will continue to investigate oceanographic applications of magnetic data.

## Data Availability

The data used to generate the figures for this study is available in Zenodo [59].

## Appendix A

### (a) Electromagnetic model

To calculate the OCIMF at the sea surface, we make use of a two-dimensional depth-integrated finite-difference model similar to that in used in [7]. This can be referred to as the static version of the thin-shell induction model (TSIM-stat) [7,8,10,36], which is based on an approximate form of the electromagnetic induction equation. The latter is constructed from Ohm’s law for a moving conductor, and three of the four Maxwell’s equations (see equation (A1)). It was shown in [10] using covariance principles from general relativity that the three Maxwell’s equations (and therefore the induction equation) are approximately invariant to the transformation to the rotating (accelerating) frame of Earth and including long periods of a day and more. (Note that the non-relativistic speeds involved could justify invariance in a Lorentz transformation connecting inertial frames, but the transformation involved here is to an accelerating frame where the Lorentz transformation does not apply. The approximate invariance found depends not just on the speeds but also the material electrical properties involved. One of the Maxwell’s equations, the one not used in the induction equation, is not even approximately invariant in the transformation to the rotating frame.) ${𝐓}_{𝛔}$ is proportional to the depth-averaged velocities, where the scaling factor is equal to a function of the vertical correlation between the conductivities and velocities (γ) as well as the ratio of the sediment conductance to the water-column conductance (λ) [43]. The quantity (1+γ)/(1+λ) is almost always between 1 and 5 in the model output we generated for this study using an ocean state estimate described below. Secondary effects of the poorly constrained sediment conductance (approx. 2000 S, can be larger) are not included but are often small compared to ocean’s conductance (approx. 20 000 S). Because we are calculating the OCIMF at the sea surface in this study, and the OCIMF is also of interest at satellite altitudes, the full implementation of three-dimensional ocean flow and conductivity heterogeneity owing to bathymetry is not crucial [12]. Self-induction is ignored because the ocean’s magnetic fields (|𝐛|∼3 nT) are much smaller than the main field (approx. 30 000 nT), and this assumption has been verified to be accurate even at the sea surface [12].

The TSIM-stat model described in more detail by [24] is based on the induction equation

(A1)
$$\begin{array}{ccc} & \begin{array}{cc} \partial 𝐁/\partial t & =\nabla \times (𝐮\times 𝐁-(\mu_{0}\sigma {)}^{-1}\nabla 𝐁), \end{array} & \end{array}$$

where 𝐁 is the Earth’s main magnetic field, t is time and $\mu_{0}$ is the vacuum magnetic permeability [10]. This model is a two-dimensional quasi-static form of the electromagnetic induction equation for a fluid moving in a rotating frame. It is discretized using finite volumes native to the ocean model (see below), and the solution is obtained through direct inversion. Its quasi-static form is appropriate for longer time scales of general ocean circulation, as opposed to tides. Because of the important of both the toroidal and poloidal components of the OCIMF [60], we account for both in our calculations. The TSIM-stat code requires the OCC (Σ, see equation (1.1)), and ${𝐓}_{𝛔}$ (see equation (1.1)) as inputs. Inputs to the TSIM-stat are obtained from the model–data synthesis described below, produced by the ECCO consortium. Its output is the OCIMF (𝐛).

### (b) Ocean state estimate

Inputs to the TSIM-stat are obtained from the latest available ECCO (ECCOv4r4), which has been used extensively for mechanistic investigation of ocean variability and is described in detail in the existing literature [30,31]. ECCOv4r4’s underlying ocean model is the Massachusetts Institute of Technology general circulation model (MITgcm [61]), configured at a nominal 1° resolution with 50 vertical levels and a partial cell representation of bottom topography [62]. Ocean mixing associated with unresolved motions is represented using standard parameterizations: the Gaspar *et al*. (1990) scheme [63] across neutral density surfaces and Redi (1982) scheme [64] along neutral density surfaces. Eddy-induced advective fluxes, intended to adiabatically rearrange tracers as a function of the slope of neutral density surfaces, are determined using the Gent and McWilliams (1990) scheme [65]. ECCOv4r4 also incorporates a dynamic/thermodynamic sea-ice model [66–68]. Atmospheric forcing is applied via bulk formulae, with the first guess atmospheric state variables taken from an atmospheric reanalysis [69].

ECCOv4r4 is constrained to fit observations through a gradient-based iterative least-squares minimization of the model–data misfit cost function. The gradient of this cost function with respect to a set of controls, comprising atmospheric forcing fields, initial conditions and ocean mixing parameters, is obtained via the adjoint model, constructed using automatic differentiation [70,71]. Appropriate control adjustments for reducing the model–data misfit are then determined using a quasi-Newton line search [72,73]. This framework accounts for model representation error and observational uncertainty in the construction of the cost function

(A2)
$$\begin{array}{ccc} & \begin{array}{cc} J & =\sum_{t=1}^{t_{f}}[𝐲(t)-𝐒\tilde{𝐱}(t){]}^{T}𝐖(t)[𝐲(t)-𝐒\tilde{𝐱}(t)], \end{array} & \end{array}$$

where $t_{f}$ is the final time step, $\tilde{𝐱}$ is the model-based estimate of the state vector 𝐱, 𝐒 is the observation matrix that relates the model state vector to observed variables (such that $𝐒\tilde{𝐱}$ is the model-based estimate of the observables 𝐲) and 𝐖 is the weight (inverse square of the uncertainty) of the observations. Both *in situ* and satellite data from a wide range of platforms were assimilated with ECCO over 1992–2017, and these optimization runs have been iterated 59 times to estimate the model parameters with which we begin our simulations.

ECCOv4r4 spans the period 1992–2017 and has been constrained to fit an extensive suite of observations, including *in situ* temperature and salinity data from profiling floats, ship-based acquisitions and gridded fields from the World Ocean Atlas, in addition to sea-surface height anomalies, mean dynamic topography, ocean-bottom pressure anomalies and sea-ice concentration from satellite. The ECCOv4r4 solution from 1993 to 2017 is used for our trend analysis of Σ, $T_{\sigma }$, the OCIMF and the model’s momentum budget (see §3c). The first available year (1992) was omitted owing to comparatively fewer altimetric constraints [74] and to avoid initialization artefacts. We did, however, use 1992 in our adjoint sensitivity experiments because we are not analysing trends for those example calculations. The electrical conductivity of seawater was calculated inline as the model ran using TEOS-10 software [75], translated to Fortran. Using the Σ and $T_{\sigma }$ fields from ECCOv4r4 as inputs to the TSIM-stat model, we calculate the OCIMF.
